# Towards Open Access

**DOI:** 10.1186/1476-4598-4-20

**Published:** 2005-06-02

**Authors:** Gregory C Ippolito, Christian Schmidt, Chhaya Das, Philip W Tucker

**Affiliations:** 1 Section of Molecular Genetics and Microbiology and Institute for Cellular and Molecular Biology, University of Texas, Austin, TX 78712, USA; 2 Molecular Cancer, BioMed Central Ltd, Middlesex House, 34-42 Cleveland Street, London W1T 4LB, UK

## 

The National Institutes of Health has issued a new policy for open access to all NIH-funded research, whereby NIH grantees are strongly encouraged to deposit their complete manuscripts and supplementary material to an Internet repository, thereby enabling the rapid dissemination of research results and the long-term archiving of scientific literature [1,2]. Entitled "Policy on enhancing public access to archived publications resulting from NIH-funded research," the Public Access Policy is a major step towards the fulfillment of a universal right to access scientific information without barriers and free of charge (i.e., Open Access). The new policy derives historically from a request made by the US House Committee on Appropriations to the National Library of Medicine in 2004 to identify potential remedies to "ensure that taxpayer-funded research remains in the public domain" and to "alleviate the restrictive trend in information technology" caused by a "dramatic rise in medical research data subscription costs" [3].

### Publish or Perish

Publications are perhaps the sole currency of scientific research--for it is publications which beget funding, and in turn, funding which begets more publications--and as such they are vitally important to the career of any research scientist. How, when, and where the research is published can be as significant as the research results themselves since the influence of a research article may only be as potent as its ability to attract an audience of readers and thereby disseminate through the field.

Indeed, the root of the word publication implies its dissemination to a *public *readership generally, and in this way the progress of science is archived in the historical record. True to this spirit, the NIH has initiated the Public Access Policy [1,2] and has created a single repository (PubMed Central, or PMC) to archive the corpus of biomedical research--past, present, and future. This Public Access Policy follows on the heels of a similar initiative in the United Kingdom last year when the House of Commons Science and Technology Committee recommended the promotion of Open Access in the UK to all publicly funded scientific research [4].

### Open Access, or How to Archive Yourself for the Ages

The NIH proposal mandates Open Access, but only to those research articles deriving in part or whole from direct costs provided by NIH grants. Nonetheless, this policy will likely apply to a major fraction of all research publications. By its own estimation, the NIH currently funds at least ten percent (65,000 articles) of all biomedical literature annually [5]. Moreover, PubMed Central will further expand due to the continuing submission (since its inception in 2000) of all final articles published in Open Access journals. To date, PubMed Central [6] archives approximately 100,000 articles from over 130 biomedical journals. Such a single repository, covering the full spectrum of research literature and freely available to the world, is revolutionary. The NIH directive, although strongly encouraged, was issued merely as a "request," and so the successful implementation of the Public Access Policy will rest on the shoulders of researchers, and traditional publishing houses, and their collective wherewithal to build the archive by willfully submitting manuscripts to the PMC.

The journal *The Proceedings of the National Academy of Sciences USA (PNAS) *has fully embraced the new NIH open-access policy. *PNAS *has in the past and will continue in the future to deposit all of its final, publisher-formatted articles into the PMC repository after a six-month embargo, regardless of the funding source. Furthermore, *PNAS *has adopted a democratic compromise by allowing authors to choose an "Open Access option" whereby the publisher-formatted final edition of their paper can be made freely available at PMC and *PNAS *immediately upon its online publication [7]. For this, *PNAS *imposes a modest surcharge ($750) that is in line with the "author pays" model used presently by most of the approximately 1500 Open Access journals [8].

Open Access already exists [9] and other traditional, subscription-based journals are invited to follow the bellwether example of *PNAS*, and at least explore the possibility of making the transition to an Open Access model of publishing. Indeed it seems that an increasing number of subscription-based journals are exploring hybrid approaches, such as that used by *PNAS*, that enable authors to offer their content online by paying publication costs for open access. But the publishing world is uniquely devoid of market forces that might expedite this transition. One need only look to the skyrocketing subscription costs (print or online) for scientific and medical journals, and the inability of institutional libraries to afford them. A study by the NIH concluded that journal prices increased during the last decade at a rate that was over 6 times inflation [10]. A recent editorial published in the *New England Journal of Medicine *acknowledges that some journal editors and publishers will perceive some aspects of the Public Access Policy as "potential threats" to their "revenue sources" [11]. In addition, some traditional publishers have assaulted Open Access journals and the NIH policy initiative with dubious arguments; the response of the Open Access community speaks for itself [1,2,12,13].

### Open Access, or How to Improve your Impact Score

A study by Thomson ISI–the company that assigns impact factors to journals–indicates that, among the 200 Open Access journals it monitors, these journals are competitive with their traditional counterparts: Open Access journals "adhere to high publishing standards, are peer reviewed comparably to other journals in their respective fields, and are cited at a level that indicates they compete favorably with similar journals in their field" [14]. One telling example is the high impact factor of the relatively new *BMC Bioinformatics *[15]. Since Open Access journals are freely available to all researchers with a connection to the Internet, the journals' impact factors are enhanced *de facto *due to the immediacy and ease with which their research articles may be retrieved, disseminated, and cited by scientific colleagues. It is this immediacy that, in part, fuels the movement towards Open Access, for such immediacy is required now more than ever given the breakneck speed of scientific progress. This is reflected in the changing landscape of scientific publishing as it transitions from print-based to electronic journals for the communication of biomedical research.

BioMed Central (BMC) and the Public Library of Science (PLoS) are two current, and successful, Open Access publishing models that provide immediate access to biomedical research articles. With high visibility and high quality articles monitored by strict peer-review standards, these publishing "houses" provide an alternative to traditional print journals and embody the ideals recently articulated in the NIH Public Access Policy. Thus, we encourage compliance with the new NIH policy and, furthermore, would suggest that authors submit their research articles to an Open Access journal, all of which archive their content in PubMed Central.

Alternatives to traditional subscription-based journals continue to gain momentum as policies in support of Open Access are made by some of the world's largest science funding agencies, namely, the NIH, the Howard Hughes Medical Institute, and the Wellcome Trust. All these agencies now provide generous budget supplements to cover the costs of publication in Open Access journals [10]. It is important to emphasize that, just as indirect costs included in NIH grant funds have long been used for financing journal subscriptions, NIH grant policies permit the use of grant funds to pay for publication costs. In fact, the NIH has made the conservative estimate that $30 million already is paid annually in direct costs to traditional print journals to defray page charges and other publication costs [5]. This sum would easily cover the author expense of 30,000 Open Access publications, if one assumes, conservatively, an average cost of $1,000 per article.

Publication models evolve over time. We have entered a very exciting period in which the scientific community can choose between two models, the traditional, subscription-based model or the Open Access model. As the number of Open Access journals and publishing houses grows, the scientific community is now in the position to `vote' for one or the other model. We firmly believe that immediate and unrestricted access to scientific information will be the gold standard for scientific publication and urge every researcher to submit their manuscripts to Open Access journals. The growing number of freely available articles, which are archived in PubMed Central, marks the trend towards Open Access.

## Competing interests

GCI, CD and PWT declare that there are no competing interests. CS is Deputy Editor of *Molecular Cancer *and receives no remuneration for his efforts. CS is exempted from *Molecular Cancer's *Article Processing Fee.

## Authors' contributions

GCI wrote and finalized this manuscript. CS, CD and PWT provided critique and suggestions. All authors read and approved the final manuscript.
